# Extremely large third-order nonlinear optical effects caused by electron transport in quantum plasmonic metasurfaces with subnanometer gaps

**DOI:** 10.1038/s41598-020-77909-y

**Published:** 2020-12-04

**Authors:** Takashi Takeuchi, Kazuhiro Yabana

**Affiliations:** Center for Computational Sciences, University of Tsukuba, Tsukuba, 305–8577 Japan

**Keywords:** Nanoscience and technology, Optics and photonics

## Abstract

In this study, a third-order nonlinear optical responses in quantum plasmonic metasurfaces composed of metallic nano-objects with subnanometer gaps were investigated using time-dependent density functional theory, a fully quantum mechanical approach. At gap distances of ≥ 0.6 nm, the third-order nonlinearities monotonically increased as the gap distance decreased, owing to enhancement of the induced charge densities at the gaps between nano-objects. Particularly, when the third harmonic generation overlapped with the plasmon resonance, a large third-order nonlinearity was achieved. At smaller gap distances down to 0.1 nm, we observed the appearance of extremely large third-order nonlinearity without the assistance of the plasmon resonance. At a gap distance of 0.1 nm, the observed third-order nonlinearity was approximately three orders of magnitude larger than that seen at longer gap distances. The extremely large third-order nonlinearities were found to originate from electron transport by quantum tunneling and/or overbarrier currents through the subnanometer gaps.

## Introduction

A plasmonic metasurface composed of periodically arrayed metallic nano-objects in two-dimensions has been demonstrated to be a useful platform for manipulating light-matter interactions. These interactions can be finely tuned over a wide range by the geometric characteristics of the nano-objects such as the object shapes, gaps (distance between the objects), and periodic patterns^1, 2^. Although metasurfaces are well established in various linear optical applications, including subdiffraction lensing^3, 4^, monochromatic or color holography^5–8^, polarization converters^9, 10^, broad bandwidth Fourier lens^11^, and energy-tailorable multifunctional thin film^12^, there has been a growing interest in their applications for nonlinear optics, such as frequency converters^13–15^, optical switching and modulation^16–18^, and others^19, 20^, in the past decade. Particularly, in recent years, a metasurface combined with multi-quantum-well semiconductor heterostuctures has attracted great attention to enhance a second-order optical nonlinearity that is expected for various applications^21, 22^.

One of the key components for achieving a high nonlinearity in a metasurface is to generate a strongly enhanced electromagnetic field in the vicinity of the nano-objects. Such an enhancement can be realized by arranging the objects to make the gap distances small so that the induced surface plasmonic charge densities are closely coupled with each other. To further strengthen the enhancement, recent experimental studies have fabricated metasurfaces with extremely small gap distances using a self-assembly approach, reaching to a subnanometer scale^23–25^. In particular, the latest report has shown that a large third-order nonlinear susceptibility can be achieved in a plasmonic metasurface composed of ligand-capped gold nanospheres with a gap distance of 0.6 nm^25^. The study used an incident light pulse whose fundamental frequency was far from the plasmonic resonance. Although the plasmon resonance usually plays a key role in the electromagnetic field enhancement, it also causes unfavorable cumulative thermo-optical effects. Using the off-resonant condition, the high enhancement originating from the large screening charge densities was realized with negligibly small heat accumulation owing to the subnanometer gaps. Such high nonlinearity achieved in metasurfaces will be crucially important for downsizing all-optical switches, as their size is inversely proportional to their nonlinear refractive index^16–20^. In this way, plasmonic metasurfaces with subnanometer gaps are expected to be ideal candidates for nonlinear optical switches that could dramatically accelerate the evolution of optical communication network systems.

In addition to the enhanced electromagnetic fields in plasmonic systems with subnanometer gaps, they also produce accompanying quantum mechanical effects. In the linear response of an isolated nanodimer system composed of two metallic nanoparticles with a small gap, quantum effects have been shown to affect the optical characteristics as previously reported in theoretical^26–30^ and experimental^31–33^ studies. The effects depend strongly on the relationship between the Fermi energy and the potential barrier at the gap. When the nanodimer is separated by a large gap distance, the Fermi energy is sufficiently lower than the potential barrier. At smaller gap distances comparable to the radius of the constituent nanoparticles, the potential barrier starts to decrease; however, the barrier height is still quite high compared to the Fermi energy. When the gap becomes smaller than 0.4 nm, the Fermi energy is approximately equal to or slightly greater than the potential barrier. In this case, electrons may cross the barrier via quantum mechanical tunneling and/or overbarrier currents through the gap. These currents produce charge transfer through the gap that suppresses the plasmonic enhancement.

In nonlinear responses, the quantum current flowing in the nanodimer has been reported to affect the electric field enhancement^34^ and the harmonic generation efficiency^35^. However, to the best of our knowledge, there have been no prior theoretical or experimental reports that discuss how such currents flowing across the gaps contribute to the nonlinearity of the metasurface with subnanometer gaps. Although there have been a few recent measurements, the gap distance was 0.6 nm in the smallest case, where the nonlinearities of the metasurface should still be solely determined by the strong optical enhancement at the gaps^25^. Therefore, it is highly intriguing to explore whether higher nonlinearity can be achieved by the currents flowing through the gaps of the metasurface.

In this study, quantum plasmonic metasurfaces composed of metallic nanospheres with subnanometer gaps were theoretically investigated through a fully quantum mechanical calculation using time-dependent density functional theory (TDDFT)^36, 37^ combined with a two-dimensional (2D) coarse-graining approach^38^ to the electromagnetism. We investigated their third-order nonlinearities for off-resonant incident pulses and clarified their dependence on the fundamental frequency and the gap distance. Our results show that, for gap distances of ≥ 0.6 nm, third-order nonlinearities monotonically increase as the gap distance decreases owing to the optical enhancement at the gap. In particular, when the frequency of the third harmonic generation overlaps with the plasmon resonance frequency, strong resonant third-order nonlinearities were observed. At smaller gap distances down to 0.1 nm, we found the appearance of extremely large third-order nonlinearity that is not assisted by the plasmon resonance. At the gap distance of 0.1 nm, the observed third-order nonlinearity is approximately three orders of magnitude larger than those at longer gap distances. It was found that the extremely large third-order nonlinearities originate from electron transport by quantum tunneling and/or overbarrier currents through the subnanometer gaps. Our findings demonstrate a new way to increase the nonlinearities of metasurfaces that should be enormously useful for downsizing all-optical switches.

## Results

### Studied system and theoretical approach

**Figure 1:**
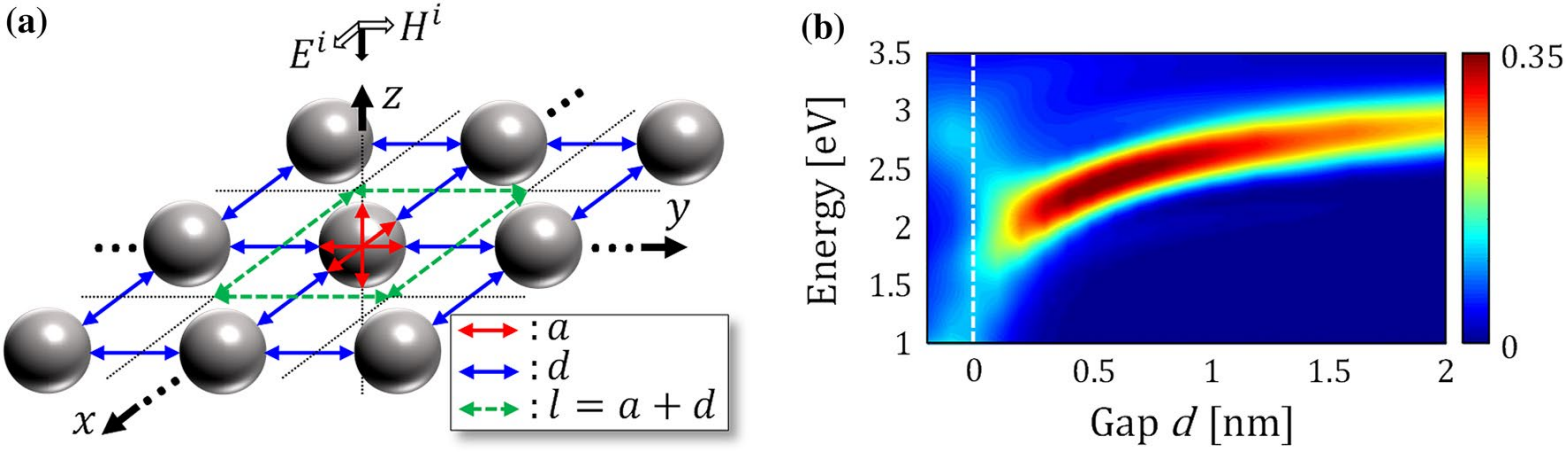
(**a**) Schematic picture of the studied metasurface composed of Na nanospheres. The parameters *a*, *d*, and *l*, represented by the red, blue, and green arrows, denote the diameter of the sphere, the gap distance, and the length of the period, respectively. (**b**) Linear optical absorption rate of the metasurfaces. The horizontal and vertical axes denote the gap distance *d* and the optical frequency, respectively. The white dashed line indicates *d* = 0 nm where the constituent spheres start to overlap geometrically.

Figure 1a displays the studied system where metallic nanospheres with a diameter *a* are periodically arrayed in the *xy* plane with a gap distance *d* and a period length *l*. The incident light of a planar pulse propagates toward the negative *z* direction with the *x*-electric and *y*-magnetic components, ${E}^{i}$ and ${H}^{i}$, respectively. The time profile of ${E}^{i}$ is described in the Supplementary Information. To treat the quantum mechanical effects with a moderate computational cost, we employed the jellium model (JM) in which ionic structures are replaced by a positive background charge of a spherical shape with a shape boundary. Although this JM includes a considerable simplification, the plasmonic motion of electrons in the nanoparticles are well described^26, 28, 39^. Previous studies reported quantitative agreement between the JM and measurements of plasmonic systems with subnanometer gaps where the quantum electron tunneling played a key role^31, 32^. In the JM, the medium is specified by the Wigner–Seitz radius, *r*_*s*_, that specifies the average charge density, *n*^+^  = ((4*π*)*r*_*s*_^3^/3)^−1^. We employed a value of *r*_*s*_ = 4.01 Bohr corresponding to the Na metal. We set *a* = 3.1 nm, where each nanosphere included 398 electrons that constitute the closed shell structure. This size is sufficiently large to ensure that the nanoparticle exhibits a well-developed plasmonic resonance^29^.

To calculate the optical responses of the metasurface, we employed the TDDFT that has been extensively used to investigate the optical properties of molecules^40^ and solids^41^ at a first-principles level. We combined the TDDFT with a 2D coarse-graining approach in which the light-matter interaction in two-dimensional materials is aptly described by coupling to the Maxwell’s equations^38^. Adiabatic and local density approximations were used for the exchange–correlation potential^42^. All the calculations were carried out using SALMON, an open-source code (https://salmon-tddft.jp/) developed in our group^43^. The Supplementary Information contains a detailed description of the adopted numerical approach.

### Linear optical response

Before discussing nonlinear optical responses, we briefly look back on the linear response of the metasurface. Figure 1b shows the linear optical absorption rate of the metasurfaces as the gap distance *d* was varied. When *d* is sufficiently large, the optical absorption shown by a bold red and yellow band appears at approximately 3 eV, which is not significantly different to the plasmon resonance of a single nanosphere. As the gap distance *d* decreases to 0.2 nm, the frequency of the absorption starts to be red-shifted, and the magnitude increases. These features originate from increasing interactions between the nanospheres. The plasmonic charge densities induced on the spheres are strongly attracted to each other, forming the bonding dipolar plasmon mode. However, at the locus of *d* ≤ 0.2 nm, the plasmon resonance rapidly decays and hybridizes into multiple plasmon modes including the bonding octopolar- and the void-plasmon modes that are caused by quantum tunneling and/or overbarrier currents flowing through the subnanometer gaps. At the locus of *d* ≤ 0 nm where the constituent spheres start to overlap geometrically, there is no potential barriers that prevent a conduction current from flowing throughout the metasurface. These trends had already been established in our previous study^44^.

### Nonlinear optical response

Next, we explored the nonlinearities in the optical response of the plasmonic metasurfaces. First, we focused on the metasurface with *d* = 2 nm where the nanospheres are sufficiently separated from each other, and the response reflects the characteristic features of a single nanosphere, as seen in Fig. 1b. In the 2D coarse-graining approach adopted here, we employed a macroscopic description in which the metasurface shown in Fig. 1a was treated as a uniform thin-film of zero-thickness^38^. In this approach, the evolution of the electric field was described by ${E}^{t}={E}^{i}-(2\pi /c)\widetilde{J}[{E}^{t}]$, where ${E}^{i}$ and ${E}^{t}$ are the incident and the macroscopic transmitted electric fields, and $\widetilde{J}[{E}^{t}]$ is the 2D macroscopic electric current density that includes nonlinear signals and is produced by the field ${E}^{t}$. The current $\widetilde{J}[{E}^{t}]$ was calculated from the microscopic electron dynamics for which we utilize the TDDFT. The boundary condition on the film, ${E}^{t}={E}^{i}+{E}^{r}$, determines the macroscopic reflected electric field, ${E}^{r}$. The details of the theory are explained in the Supplementary Information. These macroscopic reflected and transmitted electric fields, ${E}^{r}$ and ${E}^{t}$, should be observed in actual measurements.

**Figure 2:**
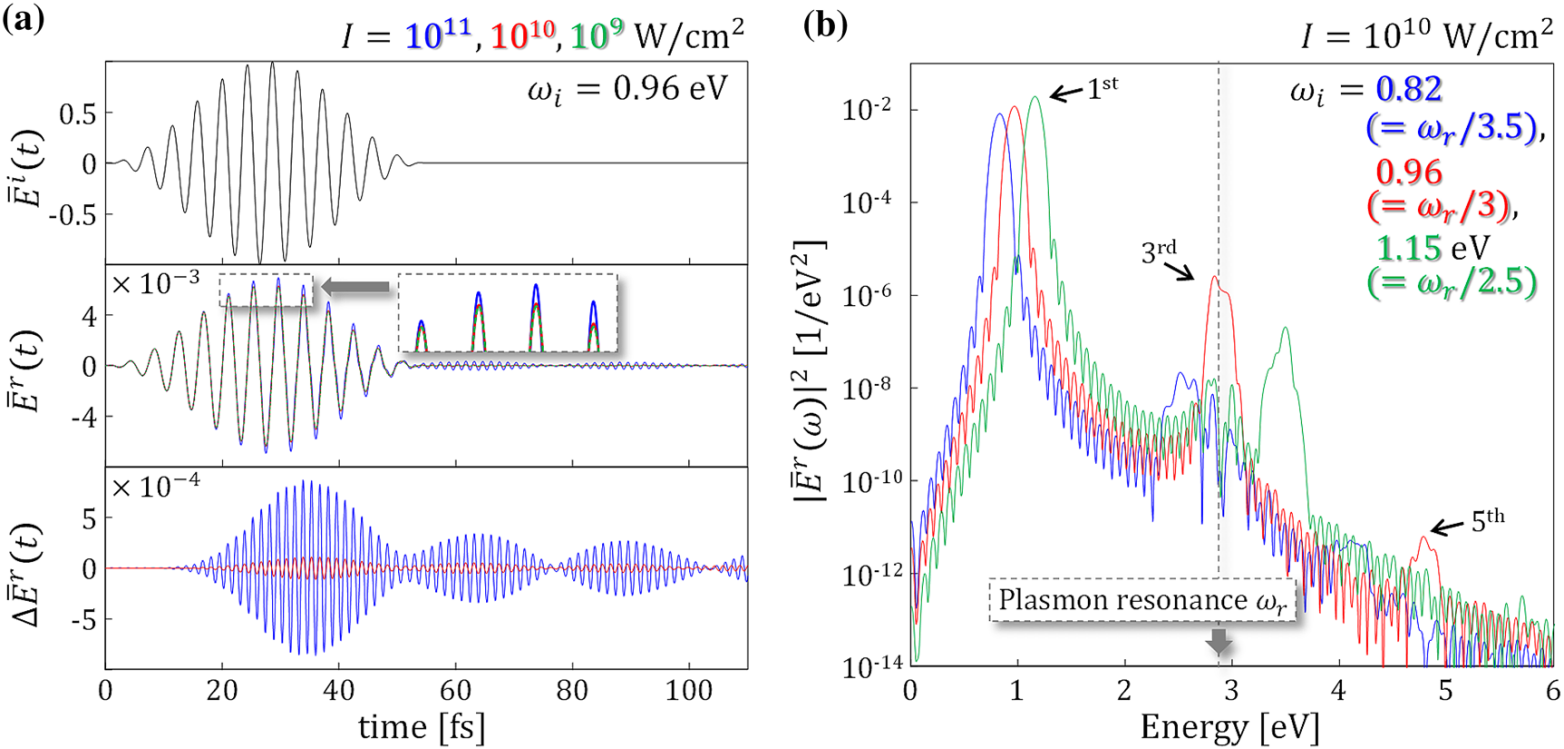
(**a**) Time-domain responses of the metasurface with the gap distance *d* = 2 nm. All electric fields are normalized by the maximum amplitude of the incident pulse, ${E}^{i}$. The fundamental frequency, *ω*_*i*_, is set to 0.96 eV. The top panel shows the normalized incident pulse $\overline{E}^{i}$ as the black line. The middle panel shows the normalized reflected electric field $\overline{E}^{r}$, where the blue, red, and green lines correspond to the three different intensities *I* = 10^11^, 10^10^, and 10^9^ W/cm^2^, respectively. The gray box shows the magnified view. The bottom panel shows the difference between each $\overline{E}^{r}$ and the lowest intensity reflection, 10^9^ W/cm^2^, $\Delta \overline{E}^{r}$. The blue and red lines represent reflection intensities of 10^11^ and 10^10^ W/cm^2^, respectively. (**b**) Power spectrum of the normalized reflected electric field |$\overline{E}^{r}$|^2^ at *I* = 10^10^ W/cm^2^. The vertical gray line indicates the frequency of the plasmon resonance, *ω*_*r*_, at *d* = 2 nm. Results for three different frequencies, *ω*_*i*_ = 0.82 (= *ω*_*r*_/3.5), 0.96 (= *ω*_*r*_/3), and 1.15 (= *ω*_*r*_/2.5) eV are plotted by the blue, red, and green lines, respectively.

Figure 2a displays the time profiles of ${E}^{i}$ and ${E}^{r}$. In all subsequent results, electric fields divided by the maximum amplitude of ${E}^{i}$ will be shown and denoted as $\overline{E}$. The full duration of the incident pulse was set to 55 fs with the envelope shaped by a cosine-squared function. The mathematical expression for ${E}^{i}$ is described in the Supplementary Information. The top panel displaying the black solid line is $\overline{E}^{i}$, where the fundamental frequency *ω*_*i*_ is set to 0.96 eV, far from the plasmon resonance at *d* = 2 nm, which is approximately 3 eV as seen in Fig. 1b. The middle panel shows $\overline{E}^{r}$ calculated for the three different incident intensities *I* = 10^11^, 10^10^, and 10^9^ W/cm^2^ that are plotted by the blue, red, and green lines, respectively. Since we applied the off-resonant incident pulse, $\overline{E}^{r}$ was much smaller than $\overline{E}^{i}$, indicating a high transparency. After *t* ≈ 20 fs, small nonlinear signals that depend on *I* were observed, as seen in the magnified box. To distinguish between the nonlinear signals more clearly, we calculated, $\Delta \overline{E}^{r}$, the difference between $\overline{E}^{r}$ and the lowest intensity reflection of 10^9^ W/cm^2^. The bottom panel shows $\Delta \overline{E}^{r}(t)$ for the two cases of *I* = 10^11^ and 10^10^ W/cm^2^, where the third harmonic generations ware clearly detected as the intensity increased.

In all subsequent results, we used a single intensity of *I* = 10^10^ W/cm^2^ that corresponds to the intensity of the pulse used in the previous experimental study on the plasmonic metasurface with subnanometer gaps^25^. To clarify the dependence on the fundamental frequency *ω*_*i*_, we examined three different *ω*_*i*_ values for the same metasurface, characterized by *d* = 2 nm. Figure 2b shows the resultant power spectrum of the normalized reflected electric field |${\overline{E}}^{r}\left(\omega \right)$|^2^, where the plasmon resonant frequency *ω*_*r*_ is indicated by the vertically drawn gray line. The blue, red, and green lines correspond to *ω*_*i*_ = 0.82 (= *ω*_*r*_/3.5), 0.96 (= *ω*_*r*_/3), and 1.15 (= *ω*_*r*_/2.5) eV, respectively. At the first harmonic generation, |$\overline{E}^{r}(\omega )$|^2^ slightly increased with *ω*_*i*_ because the frequency comes slightly closer to the plasmon resonance *ω*_*r*_. In contrast, for the third harmonic generation appearing around 3 eV, the highest nonlinearity was achieved at *ω*_*i*_ = 0.96 eV. This is because the third-order signal appears closely to *ω*_*r*_ and thus is plasmonically enhanced. There is a time-delay in the third-order harmonic generation with respect to the incident and the reflected fields, as seen by comparing the top, middle, and bottom panels of Fig. 2a, due to the inherent time requirement for resonant enhancement. The enhanced nonlinearity assisted by the plasmon resonance was also reported in the previous study that dealt with an isolated nanodimer theoretically^31^. Finally, we note that the fifth harmonic generation is visible only at *ω*_*i*_ = 0.96 eV.

### Dependence on gap-size

**Figure 3:**
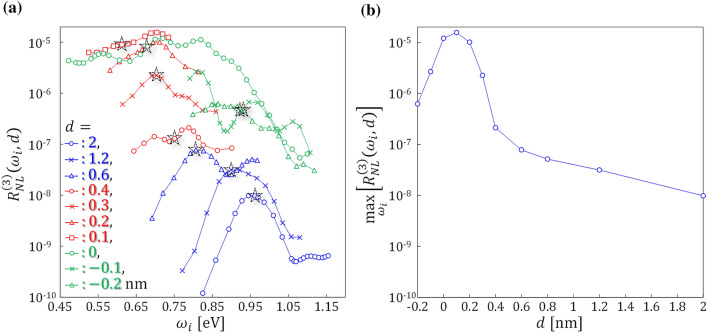
(**a**) Fundamental frequency dependence of the third harmonic generation component of the nonlinear reflection rate ${R}_{NL}^{\left(3\right)}$, whose definition is described in the main text. Results of *d* = 2 to − 0.2 nm are shown by different symbols with colored lines. The black pentacles indicate the conditions at which *ω*_*i*_ = *ω*_*r*_/3 is satisfied for each *d*. At *d* = 0 nm, the pentacle is not shown. (**b**) Gap distance dependence of max_*ωi*_[${R}_{NL}^{\left(3\right)}\left({\omega }_{i},d\right)$], the maximum value of ${R}_{NL}^{\left(3\right)}$ at each *d*.

We now move on to the main subject of the present study, clarifying the third-order nonlinearities of metasurfaces with various subnanometer gaps. To quantify the third-order nonlinear efficiency, we introduce a quantity, ${R}_{NL}^{(3)}\left({\omega }_{i},d\right)=\left[{\int }_{2.5{\omega }_{i}}^{3.5{\omega }_{i}}{\left|{E}^{r}\left(\omega \right)\right|}^{2}d\omega \right]/\left[{\int }_{0}^{\infty }{\left|{E}^{i}\left(\omega \right)\right|}^{2}d\omega \right]$, the detailed definition of which is described in the Supplementary Information. In simple terms, ${R}_{NL}^{(3)}$ indicates the nonlinear reflectivity caused by third harmonic generation for the case with a gap distance *d* and the incident pulse with a fundamental frequency *ω*_*i*_. Figure 3a summarizes the resultant ${R}_{NL}^{(3)}$ for gap distances from − 0.2 to 2 nm and the fundamental frequencies *ω*_*r*_/3.5 < *ω*_*i*_ < *ω*_*r*_/2.5, where *ω*_*r*_ is the plasmon resonant frequency. When the fundamental frequency satisfies *ω*_*i*_ = *ω*_*r*_/3, it is marked in Fig. 3 by a black pentacle. At *d* = 0 nm, *ω*_*i*_ is widely sampled from 0.47 to 1.1 eV, and the pentacle is not added to this case because the plasmon resonance is hardly distinguished, as seen in Fig. 1b. Figure 3a allows us to find three distinctive trends that are indicated by the different colored lines. The first trend is indicated by the blue lines representing *d* = 2, 1.2, and 0.6 nm. Here, the peaks appear close to the pentacles. The peak values gradually increase and the peak frequencies are red-shifted as the gap size decreases. These trends are in accordance with the linear responses shown in Fig. 1b, indicating that the large third-order nonlinearities are assisted by the plasmonic resonance of the bonding dipolar mode. The second trend is indicated by the red lines consisting of *d* = 0.4, 0.3, 0.2, and 0.1 nm. In this group, the peak frequencies do not coincide with the pentacle except for the *d* = 0.3 nm case. At the gap distance of *d* = 0.1 nm, the peak appears around 0.7 eV. It is noteworthy that the third-order nonlinearity at *d* = 0.1 nm is three orders of magnitude larger than ${R}_{NL}^{(3)}$ at the longest gap distance case of *d* = 2 nm. The last trend is indicated by the green lines consisting of *d* = 0, − 0.1, and − 0.2 nm. Here, the constituent nanospheres start to overlap geometrically. The results shown here assume that *ω*_*r*_ for *d* = − 0.1 and − 0.2 nm is given by the upper branch, seen in the top left region of Fig. 1b. These green lines show a rapid decay of ${R}_{NL}^{(3)}$ with decreasing *d*.

To provide a clear measure of the gap distance dependence of the observed third-order nonlinearities, we introduce a new quantity, max_*ωi*_[${R}_{NL}^{\left(3\right)}\left({\omega }_{i},d\right)$], by taking the maximum ${R}_{NL}^{(3)}$ observed for varying *ω*_*i*_ at each *d*. The result is shown in Fig. 3b. It indicates that the gap distance dependence of the nonlinearity can be categorized into two regions; one is from *d* = 0.6 to 2 nm where max[${R}_{NL}^{(3)}$] monotonically increases as the gap distance is reduced, the other is from *d* = − 0.2 to 0.4 nm where max[${R}_{NL}^{(3)}$] shows a prominent maximum at *d* = 0.1 nm. To quantify the observed extremely large third-order nonlinearity of the studied metasurfaces, they were compared to the nonlinearity of a SiO_2_ thin film which is conventionally used in all-optical switches^25^. As described in the Supplementary Information, we estimated the ${R}_{NL}^{(3)}$ of a SiO_2_ thin film with the same thickness, *a*, as the present metasurface. The value was calculated to be $1.43\times {10}^{-13}$, eight orders of magnitude lower than the studied metasurface.

### The origin of high nonlinearity

**Figure 4:**
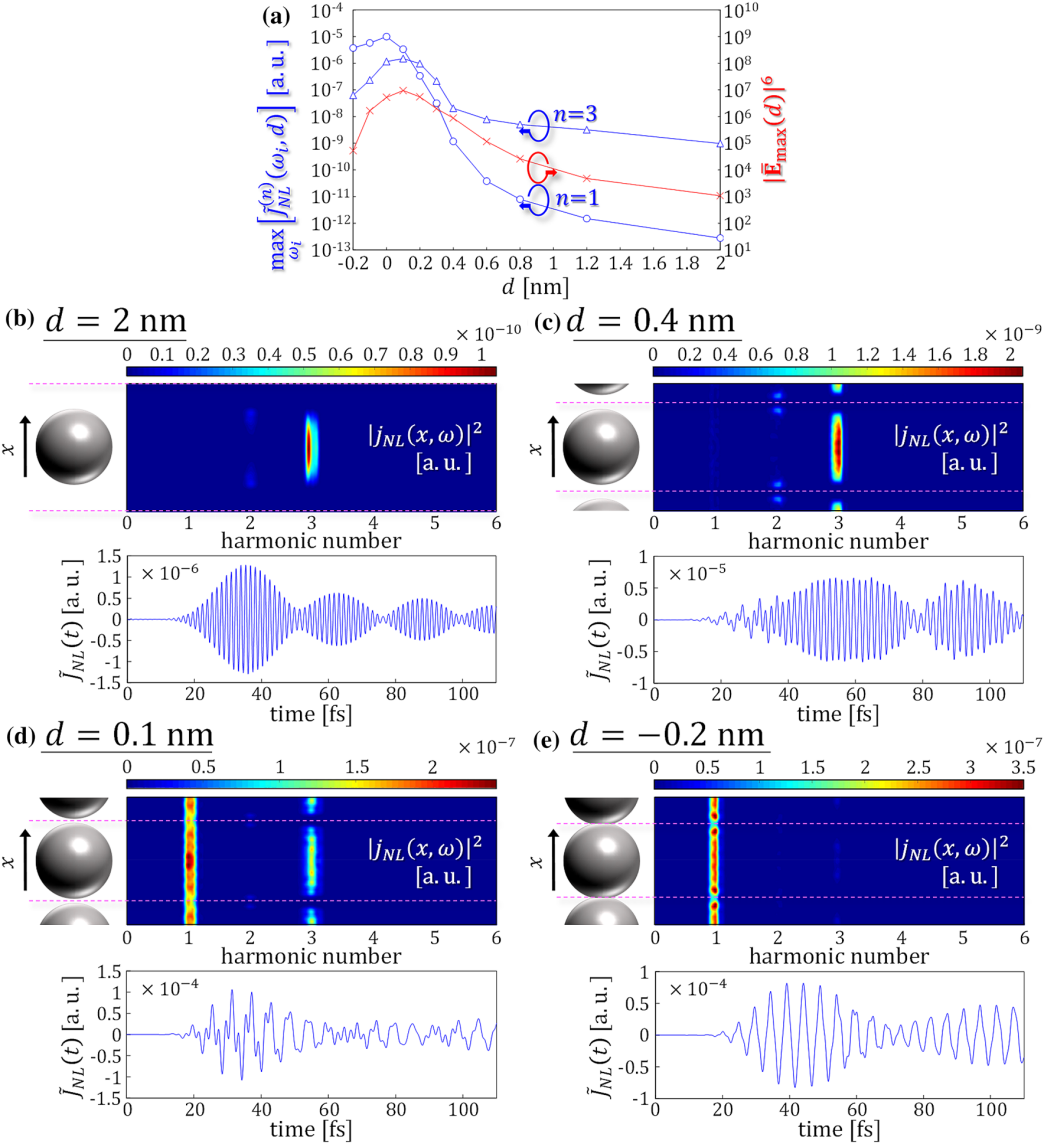
(**a**) Gap distance dependence of max_*ωi*_[${\widetilde{J}}_{NL}^{\left(n\right)}\left({\omega }_{i},d\right)$] (blue lines, left axis) and ${\left|{\overline{\mathbf{E}}}_{\mathrm{max}}\left(d\right)\right|}^{6}$ (red line, right axis). Their definitions are given in the main text. (**b**–**e**) Nonlinear two-dimensional electric current density ${\widetilde{J}}_{NL}$ (lower panels) and spatio-frequency distribution of the microscopic nonlinear electric current density ${j}_{NL}$ (upper panels) are shown for *d* = 2, 0.4, 0.1 and − 0.2 nm, respectively. The fundamental frequency *ω*_*i*_ is set to the same value as that used to calculate max_*ωi*_[${R}_{NL}^{\left(n\right)}\left({\omega }_{i},d\right)$] in Fig. 3b. In the upper panels, the vertical axes denote the *x*-axis of the metasurface which is parallel to the polarization direction of the incident pulse. The horizontally drawn pink dashed lines mark off the periodic length *l* along the *x* direction.

To clarify the physical origin of the different features observed for gap distances above and below *d* = 0.6 nm in Fig. 3b, we examined the nonlinear electric current density flowing in the metasurface. In the 2D coarse-graining approach employed here, the reflected field ${E}^{r}\left(t\right)$ is equivalent to the 2D macroscopic electric current density $\widetilde{J}(t)$ up to a constant factor, ${E}^{r}=-(2\pi /c)\widetilde{J}[{E}^{t}]$, and $\widetilde{J}(t)$ is given from the microscopic current density $j\left(x,y,z,t\right)$ by $\widetilde{J}(t)=\int \int \left(dxdy/{l}^{2}\right)\int dzj\left(x,y,z,t\right)$. To investigate the physical mechanism that produces the nonlinear behavior, we introduce the nonlinear current density defined as ${j}_{NL}(x,t)=\int \left(dy/{l}^{2}\right)\int dz\left[j\left(x,y,z,t\right)-\sqrt{I/{I}_{L}}{j}_{L}(x,y,z,t)\right]$, where ${j}_{L}$ is the linear current density that is calculated using the incident pulse with the same time profile and sufficiently weak intensity ${I}_{L}$. In practice, we use ${I}_{L}$ = 10^9^ W/cm^2^ to calculate the linear current. Likewise, we define the nonlinear 2D current density, $\widetilde{J}_{NL}\left(t\right)$ by integrating ${j}_{NL}(x,t)$ over *x*. To investigate nonlinear optical responses that appear in different frequency regions, we introduce the spectrally divided nonlinear 2D current density, ${\widetilde{J}}_{NL}^{(n)}\left({\omega }_{i},d\right)={\int }_{(n-0.5){\omega }_{i}}^{(n+0.5){\omega }_{i}}{\left|{\widetilde{J}}_{NL}\left(\omega \right)\right|}^{2}d\omega$. For *n* = 3, this quantity is proportional to ${R}_{NL}^{(3)}$ in Fig. 3b. Similarly as in Fig. 3b, we took the maximum of ${\widetilde{J}}_{NL}^{(n)}$ for ${\omega }_{i}$ at each *d*; the result is presented in Fig. 4a where the blue lines with the circles and the triangles correspond to *n* = 1 and 3, respectively. The max[${\widetilde{J}}_{NL}^{(3)}$] shows similar behavior to ${R}_{NL}^{(3)}$. Since ${\widetilde{J}}_{NL}$ starts from the third-order to the electric field, we show ${\left|{\overline{\mathbf{E}}}_{\mathrm{max}}\right|}^{6}$ by the red line with the crosses for reference, where $\overline{\mathbf{E}}_{\mathrm{max}}$ denotes the maximum electric field sampled on the *xy* plane including the center of mass of the nanospheres, normalized by the amplitude of the incident pulse. For the cases where *d* ≥ 0.6 nm, both max[${\widetilde{J}}_{NL}^{(n)}$] with *n* = 1 and 3 increase as *d* decreases, and are approximately proportional to ${\left|{\overline{\mathbf{E}}}_{\mathrm{max}}\right|}^{6}$. The magnitude of max[${\widetilde{J}}_{NL}^{(n)}$] with *n* = 3 is much larger than that of *n* = 1, owing to the resonant enhancement of the *n* = 3 component. The maximum electric field appears at the surfaces of the nanospheres, and the interaction of the induced charges at the surfaces enhances ${\left|{\overline{\mathbf{E}}}_{\mathrm{max}}\right|}^{6}$ as the gap distance decreases. At gap distances below 0.6 nm, where the extremely large third-order nonlinearities are obtained, the increase of the ${\left|\overline{\mathbf{E}}_{\mathrm{max}}\right|}^{6}$ is no longer responsible for the increase of the max[${\widetilde{J}}_{NL}^{(n)}$]. It was observed that the nonlinear current component for *n* = 1, max[${\widetilde{J}}_{NL}^{(1)}$], rapidly increased and became comparable to the component for *n* = 3. This indicates that the enhancement is no longer caused by the resonant effect.

To elucidate the behavior causing the extremely large third-order nonlinearities, we focused on the temporal and spatial distribution of the nonlinear electric current density. The upper panels of Fig. 4b–e illustrate the spatio-frequency distribution of the nonlinear microscopic electric current density ${\left|{j}_{NL}\left(x,{\omega }\right)\right|}^{2}$. The fundamental frequency, *ω*_*i*_, is set to the value that gives max[${\widetilde{J}}_{NL}^{(3)}$] for the metasurfaces with *d* = 2, 0.4, 0.1, and − 0.2 nm. The vertical axis is the *x*-axis of the metasurface that is parallel to the polarization direction of the incident pulse. The horizontally drawn pink dashed lines mark off the periodic length *l* with the left schematics showing the constituent nanospheres. The lower panels indicate the nonlinear 2D macroscopic electric current density ${\widetilde{J}}_{NL}\left(t\right)$. In Fig. 4b, for *d* = 2 nm, ${\widetilde{J}}_{NL}\left(t\right)$ solely consists of the plasmonically assisted third harmonic generation whose spatial distribution is fully confined to the sphere, as seen from the distribution of ${\left|{j}_{NL}\left(x,\omega \right)\right|}^{2}$. This is explained by the position of the Fermi energy which is much lower than the potential barriers at the gaps. Although small second harmonic components are visible in ${j}_{NL}$ around the edge of spheres, they vanish after the spatial integration because the components have opposite signs to each other. Figure 4c displays the case of *d* = 0.4 nm, where the potential barrier is slightly higher than the Fermi energy. The nonlinear current is still mainly composed of the third harmonic component. The amplitude is larger than that of the *d* = 2 nm case owing to the stronger electric field enhancement at the gaps. It was noted that a slight nonlinear component of the fundamental frequency is visible in ${\widetilde{J}}_{NL}$ at 10 ≤ *t* ≤ 30 fs. We confirmed that this weak signal is also seen in ${\left|{j}_{NL}\left(x,\omega \right)\right|}^{2}$ flowing across the gaps by quantum mechanical tunneling, although it cannot be seen on the color scale in the upper panel. As seen in Fig. 4d, ${\widetilde{J}}_{NL}\left(t\right)$ at *d* = 0.1 nm shows drastic changes compared to the above-mentioned trends at larger gap distances. At this distance, the Fermi energy is slightly higher than the potential barriers, changing the mechanism of the current from tunneling to overbarrier. The nonlinear current includes both *n* = 1 and *n* = 3 components and propagates through the subnanometer gaps. As seen in Figs. 3b and 4a, this gap distance produced the largest third-order nonlinearity. At *d* = − 0.2 nm shown in Fig. 4e, the third harmonic generation current rapidly decreases. Here, the tunneling and/or the overbarrier currents are unified into a conduction one flowing throughout the metasurface because nanospheres are directly connected. We noted that the magnitude of the nonlinear current decreases compared to the case of *d* = 0.1 nm. From these observations, we conclude that the extremely large nonlinearity is caused by electron transport through the gaps via the tunneling and/or the overbarrier mechanisms.

While performing this study, we used various approximations. They are outlined below in conjunction with the limitations of this study. Since we assume the Na nanospheres described by the JM, it does not include any *d*-electron effects that appear in typical plasmonic materials such as the noble metals. Therefore, in our simulation, the nonlinearity caused by the *d*-electrons is ignored. Moreover, the JM ignores the ionic structure of metallic nanoparticles that may cause strong electric-field enhancements at the apexes of clusters, known as the lightning rod effect^29^.

Although we have focused on very small nanospheres with a diameter of *a* = 3.1 nm, actual plasmonic nanoparticles used widely in measurements are larger in size, reaching 10–100 nm^1, 2^. Furthermore, despite the great advantage that the optical characteristics of metasurfaces can be finely tuned over a wide range by the shape of the nanoparticles, this study remained limited to the elementary geometry of spheres. These geometrical differences would affect electron tunneling and/or overbarrier currents, modifying the nonlinearities. To explore such effects, one prospective candidate method is quantum hydrodynamic theory (QHT) that can describe the nonlinear light-matter interaction of plasmonic systems^45–47^. In particular, a recently proposed QHT study has revealed that it can be directly derived from the TDDFT with the JM, demonstrating good agreement in the linear response regime^47^. Nevertheless, the computational cost of the QHT is significantly lower than the TDDFT because the QHT is an orbital free approach. Therefore, the QHT is expected to allow investigations of much larger systems with various geometries. We consider that the application of the QHT to metallic metasurfaces and the comparison with the TDDFT results in the nonlinear regime is an important topic for a future study.

## Discussion

In conclusion, we have presented a theoretical investigation of third-order nonlinearities in quantum plasmonic metasurfaces with subnanometer gaps, mainly focused on third harmonic generation. Nonlinear optical responses of a metasurface composed of metallic nanospheres have been examined using the TDDFT with the JM, where an off-resonant incident pulse ensuring negligibly small cumulative thermo-optical effects was employed. We have calculated the third-order nonlinearities as functions of the fundamental frequency of the pulse and the gap distance of the metasurface. It has been shown that, for gap distances of ≥ 0.6 nm, the third-order nonlinearities monotonically increase as the gap distance decreases. This is caused by enhancement of the screening charge densities that are induced at the interfaces of the nanospheres. When the frequency of the third harmonic generation overlaps with the linear plasmon resonance, large third-order nonlinearities are observed. At further smaller gap distances down to 0.1 nm, we find the appearance of the extremely large third-order nonlinearity that is not assisted by the plasmon resonance. In particular, at the gap distance of 0.1 nm, we have achieved a third-order nonlinearity three orders of magnitude larger than that of the longest gap distance case. The extremely large third-order nonlinearities originate from electron transport by quantum tunneling and/or overbarrier currents through the subnanometer gaps. At gap distances of *d* ≤ 0 nm where the spheres geometrically overlap, the tunneling and/or the overbarrier currents are unified into a usual conduction current that flows throughout the metasurface. In that case, the third-order nonlinearities were observed to decrease. Our findings suggest a new way to increase the nonlinearity of metasurfaces, which is expected to be enormously useful for downsizing all-optical switches.

## Supplementary information

Supplementary Information.

## References

[1] Meinzer N, Barnes WL, Hooper IR (2014). Plasmonic meta-atoms and metasurfaces. Nat. Photonics.

[2] Choudhury SM (2018). Material platforms for optical metasurfaces. Nanophotonics.

[3] Aieta F (2012). Aberration-free ultrathin flat lenses and axicons at telecom wavelengths based on plasmonic metasurfaces. Nano Lett..

[4] Khorasaninejad M (2016). Metalenses at visible wavelengths: Diffraction-limited focusing and subwavelength resolution imaging. Science.

[5] Chen WT (2014). High-efficiency broadband meta-hologram with polarization-controlled dual images. Nano Lett..

[6] Zheng G (2015). Metasurface holograms reaching 80% efficiency. Nat. Nanotechnol..

[7] Huang YW (2015). Aluminum plasmonic multicolor meta-hologram. Nano Lett..

[8] Li X (2016). Multicolor 3D meta-holography by broadband plasmonic modulation. Sci. Adv..

[9] Yu N (2012). A broadband, background-free quarter-wave plate based on plasmonic metasurfaces. Nano Lett..

[10] Ding F, Wang Z, He S, Shalaev VM, Kildishev AV (2015). Broadband high-efficiency half-wave plate: A supercell-based plasmonic metasurface approach. ACS Nano.

[11] Liu W (2018). Metasurface enabled wide-angle Fourier lens. Adv. Mater..

[12] Liu W (2019). Energy-tailorable spin-selective multifunctional metasurfaces with full fourier components. Adv. Mater..

[13] Suchowski H (2013). Phase mismatch-free nonlinear propagation in optical zero-index materials. Science.

[14] Celebrano M (2015). Mode matching in multiresonant plasmonic nanoantennas for enhanced second harmonic generation. Nat. Nanotechnol..

[15] Grinblat G, Li Y, Nielsen MP, Oulton RF, Maier SA (2016). Enhanced third harmonic generation in single germanium nanodisks excited at the anapole mode. Nano. Lett..

[16] Wurtz GA (2011). Designed ultrafast optical nonlinearity in a plasmonic nanorod metamaterial enhanced by nonlocality. Nat. Nanotechnol..

[17] Ren M (2011). Nanostructured plasmonic medium for terahertz bandwidth all-optical switching. Adv. Mater..

[18] Harutyunyan H (2015). Anomalous ultrafast dynamics of hot plasmonic electrons in nanostructures with hot spots. Nat. Nanotechnol..

[19] Li G, Zhang S, Zentgraf T (2017). Nonlinear photonic metasurfaces. Nat. Rev. Mater..

[20] Reshef O, Leon ID, Alam MZ, Boyd RW (2019). Nonlinear optical effects in epsilon-near-zero media. Nat. Rev. Mater..

[21] Lee J (2014). Giant nonlinear response from plasmonic metasurfaces coupled to intersubband transitions. Nature.

[22] Qian H (2019). Large optical nonlinearity enabled by coupled metallic quantum wells. Light Sci. Appl..

[23] Fontana J (2016). Linear and nonlinear optical characterization of self-assembled, large-area gold nanosphere metasurfaces with sub-nanometer gaps. Opt. Express..

[24] Doyle D (2018). Tunable subnanometer gap plasmonic metasurfaces. ACS Photo..

[25] Menezes LDS (2019). Large third-order nonlinear susceptibility from a gold metasurface far off the plasmonic resonance. J. Opt. Soc. Am. B.

[26] Zuloaga J, Prodan E, Nordlander P (2009). Quantum description of the plasmon resonances of a nanoparticle dimer. Nano Lett..

[27] Mao L, Li Z, Wu B, Xu H (2009). Effects of quantum tunneling in metal nanogap on surface-enhanced Raman scattering. Appl. Phys. Lett..

[28] Esteban R, Borisov AG, Nordlander P, Aizpurua J (2012). Bridging quantum and classical plasmonics with a quantum-corrected model. Nat. Commun..

[29] Barbry M (2015). Atomistic near-field nanoplasmonics: Reaching atomic-scale resolution in nanooptics. Nano Lett..

[30] Varas A, García-González P, Feist J, García-Vidal FJ, Rubio A (2016). Quantum plasmonics: From jellium models to ab initio calculations. Nanophotonics.

[31] Scholl JA, García-Etxarri A, Koh AL, Dionne JA (2012). Observation of quantum tunneling between two plasmonic nanoparticles. Nano Lett..

[32] Savage KJ (2012). Revealing the quantum regime in tunnelling plasmonics. Nature.

[33] Scholl JA (2016). Evolution of plasmonic metamolecule modes in the quantum tunneling regime. ACS Nano.

[34] Marinica DC, Kazansky AK, Nordlander P, Aizpurua J, Borisov AG (2012). Quantum plasmonics: Nonlinear effects in the field enhancement of a plasmonic nanoparticle dimer. Nano Lett..

[35] Aguirregabiria G (2018). Role of electron tunneling in the nonlinear response of plasmonic nanogaps. Phys. Rev. B.

[36] Runge E, Gross EKU (1984). Density-functional theory for time-dependent systems. Phys. Rev. Lett..

[37] Ullrich CA (2012). Time-Dependent Density-Functional Theory Concepts and Applications.

[38] Yamada S, Noda M, Nobusada K, Yabana K (2018). Time-dependent density functional theory for interaction of ultrashort light pulse with thin materials. Phys. Rev. B.

[39] Brack M (1993). The physics of simple metal clusters: Self-consistent jellium model and semiclassical approaches. Rev. Mod. Phys..

[40] Yabana K, Bertsch GF (1996). Time-dependent local-density approximation in real time. Phys. Rev. B.

[41] Bertsch GF, Iwata JI, Rubio A, Yabana K (2000). Real-space, real-time method for the dielectric function. Phys. Rev. B.

[42] Perdew JP, Zunger A (1981). Self-interaction correction to density-functional approximations for many-electron systems. Phys. Rev. B.

[43] Noda M (2019). SALMON: Scalable Ab-initio light-matter simulator for optics and nanoscience. Comput. Phys. Commun..

[44] Takeuchi T, Noda M, Yabana K (2019). Operation of quantum plasmonic metasurfaces using electron transport through subnanometer gaps. ACS Photon..

[45] Ciracì C, Poutrina E, Scalora M, Smith DR (2012). Second-harmonic generation in metallic nanoparticles: Clarification of the role of the surface. Phys. Rev. B.

[46] Toscano G (2015). Resonance shifts and spill-out effects in self-consistent hydrodynamic nanoplasmonics. Nat. Commun..

[47] Ciracì C (2017). Current-dependent potential for nonlocal absorption in quantum hydrodynamic theory. Phys. Rev. B.

