# Supplementary material for: Extremely large third-order nonlinear optical effects caused by electron transport in quantum plasmonic metasurfaces with subnanometer gaps
Source: Sci Rep. 2020 Dec 4;10:21270. doi: 10.1038/s41598-020-77909-y (PMC7718924; doi:10.1038/s41598-020-77909-y)
Supplement: Supplementary file 1 — Supplementary Information. [file 41598_2020_77909_MOESM1_ESM.pdf]

Supplementary Information

Extremely large third-order nonlinear optical effects caused by electron transport in quantum plasmonic metasurfaces with subnanometer gaps

Takashi Takeuchi and Kazuhiro Yabana

This Supplementary Information gives detailed theoretical description of the TDDFT with the 2D coarse-graining approach and the perturbative theory to estimate the nonlinearity of a SiO_2 thin film.

1. Theory on the nonlinear optical responses of metasurfaces

We summarize the theory to calculate the nonlinear optical responses of metasurfaces adopting 2D course graining approximation[1]. The atomic unit is used in this Supplementary Information.

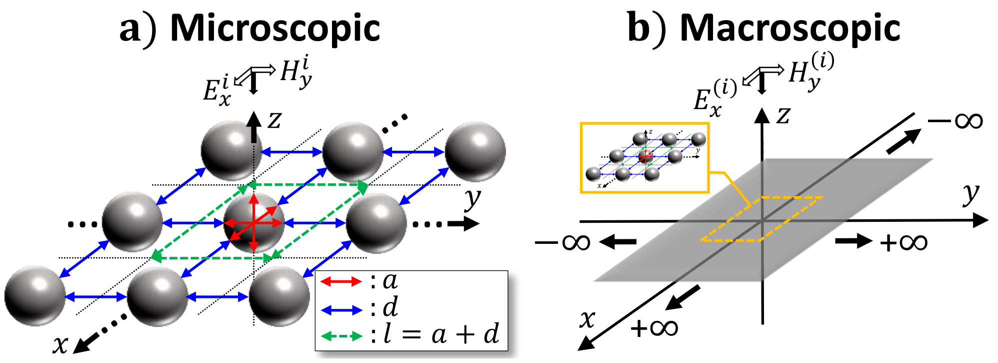

Figure 1. Studied metasurface system. The incident light is a planar pulse with E_x^i and H_y^i components that propagate along the negative z direction. (a) Microscopic view, whereby a, d, and l, are drawn using the red, blue, and green arrows, respectively, and denote the diameters of the spheres, gap distances, and the lengths of the period, respectively. (b) Macroscopic view in which we assume that the metasurface is regarded as a uniform thin film in the xy plane with an ignorable thickness in the z-direction.

We depict a microscopic picture of the metasurface in Figure 1(a) where metallic nanospheres with the diameter a are periodically arrayed on the xy plane with the gap distance d and the period length l. The electronic motion in the nanospheres are described using the

time-dependent density functional theory (TDDFT) with the jellium model as explained in the next section. We consider an incident planar pulse with E_x^i and H_y^i components propagating along the negative *z* direction. The time profile of the incident pulse E_x^i is described as

$$E_x^i\,(t) = F\cos^2\left[\frac{\pi}{T}\left(t-\frac{T}{2}\right)\right]\sin\omega_i t \quad (0 < t < T), \tag{1}$$

where F, T, and ω_i are the amplitude, pulse-duration, and fundamental frequency of the pulse, respectively. In the main text, *F* is determined from the pulse intensity *I* that is set to 10^{10} W/cm^2 unless otherwise noted while T is fixed to 55 fs, and ω_i is set far from the frequency of the plasmon resonance ω_r.

Conduction electrons in the spheres are excited by the incident pulse, and the microscopic electric current density $\mathbf{j}(\mathbf{r},t)$ appears in the spheres. If the diameter of the nanospheres is sufficiently small compared with the incident wavelength, we can safely apply the 2D course graining approximation in which the metasurface is regarded as a uniform thin film macroscopically with an ignorable thickness. We introduce the macroscopic electric current density $\mathbf{J}(\mathbf{r},t)$ of the film by

$$\mathbf{J}(\mathbf{r},t) \approx \delta(z)\tilde{\mathbf{J}}(t), \tag{2}$$

where $\tilde{\mathbf{J}}(t)$ is the coarse-grained 2D electric current density which is related to the microscopic current density $\mathbf{j}(\mathbf{r},t)$ by

$$\tilde{\mathbf{J}}(t) = \int\int\frac{dxdy}{l^2}\int dz\,\mathbf{j}(\mathbf{r},t), \tag{3}$$

where the integrations are carried out over the 2D unit cell area in the xy plane and over the z-direction. This $\tilde{\mathbf{J}}$ is parallel to the x direction owing to the symmetry of the system. By using $\tilde{\mathbf{J}}$, the following relations on the film are derived from the Maxwell's equations.

$$\mathbf{E}^t(t) = \mathbf{E}^i(t) + \mathbf{E}^r(t) = \mathbf{E}^i(t) - \frac{2\pi}{c}\tilde{\mathbf{J}}(t), \tag{4}$$

where $\mathbf{E}^t$ and $\mathbf{E}^r$ are the transmitted and reflected electric fields in the macroscopic scale. To complete the formalism, we need to connect the course-grained 2D electric current density $\tilde{\mathbf{J}}(t)$ with the macroscopic electric field on the thin film that is equal to $\mathbf{E}^t(t)$. The relevant relation will be given in the next section.

To quantify the nonlinear signals, we introduce the following reflectance rate R for the case of the gap distance *d* and the incident pulse with the fundamental frequency ω_i and the intensity I,

$$R(\omega_i, d, I) = R_L^{(1)}(\omega_i, d) + \sum_{n=1}^{\infty} R_{NL}^{(n)}(\omega_i, d, I), \tag{5}$$

$$R_L^{(1)}(\omega_i, d) = \frac{\int_{0.5\omega_i}^{1.5\omega_i} \left|E_x^r(\omega, d, I_L)\right|^2 d\omega}{\int_0^{\infty} \left|E_x^i(\omega, I_L)\right|^2 d\omega}, \tag{6}$$

$$R_{NL}^{(n)}(\omega_i, d, I) = \frac{\int_{(n-0.5)\omega_i}^{(n+0.5)\omega_i} \left|E_x^r(\omega, d, I)\right|^2 d\omega}{\int_0^{\infty} \left|E_x^i(\omega, I)\right|^2 d\omega} - \delta_{n1} R_L^{(1)}(\omega_i, d), \tag{7}$$

where $R_L^{(1)}$ is the linear reflection obtained from the sufficiently low intensity I_L while $R_{NL}^{(n)}$ denotes the *n*-th order nonlinear reflection. In the main text, we calculated $R_{NL}^{(3)}$ to explore the third-order harmonic generation in the reflected wave.

2. TDDFT

For the quantum mechanical description of the electron dynamics in the metasurface, we employ the time-dependent density functional theory (TDDFT) with the jellium model. It provides relation between the 2D electric current density, $\tilde{\mathbf{J}}(t)$, and the macroscopic eletric field on the film, $\mathbf{E}^t(t)$, that appear in Eq. (4).

According to the TDDFT, electron motion is governed by the following time-dependent Kohn-Sham equation with a periodic boundary condition in the x and y directions, and an isolated boundary condition in the z direction,

$$i\frac{\partial u_{n\mathbf{k}}(\mathbf{r},t)}{\partial t} = \left[\frac{1}{2}\left(-i\nabla + \mathbf{k} + \frac{1}{c}\mathbf{A}(t)\right)^2 - \phi(\mathbf{r},t) + V_{\mathrm{XC}}(\mathbf{r},t)\right]u_{n\mathbf{k}}(\mathbf{r},t), \qquad (8)$$

where $u_{n\mathbf{k}}$, $\mathbf{k}$, and V_{XC}, represent the Bloch orbitals, 2D crystalline momentum vector, and exchange correlation potential, respectively. $\mathbf{A}$ and ϕ are the vector and scalar potentials, respectively. In the spherical jellium model, the following charge density ρ_{jm} with the Wigner-Seitz radius r_s is assumed in the unit cell:[2–3]

$$\rho_{\mathrm{jm}}(\mathbf{r}) = n^{+}\theta\left(\frac{a}{2} - r\right), \qquad (9)$$

$$n^{+} = \left(\frac{4\pi r_s^3}{3}\right)^{-1}, \qquad (10)$$

$$\frac{a}{2} = \left(\frac{3}{4\pi}\frac{N_e}{n^{+}}\right)^{\frac{1}{3}}, \qquad (11)$$

where N_e is the number of electrons of the metallic nanosphere. In the 2D course graining approximation, the vector potential $\mathbf{A}(t)$ is determined by the macroscopic transmitted electric field $\mathbf{E}^t(t)$ on the metasurface as

$$\mathbf{A}(t) = -c\int dt\,\mathbf{E}^t(t). \qquad (12)$$

The scalar potential $\phi(\mathbf{r},t)$ is periodic in xy -plane and vanishes as $|z| \to \infty$. It satisfies the following Poisson equation with the charge densities $\rho(\mathbf{r},t)$ and $\rho_{\mathrm{jm}}(\mathbf{r})$:

$$\Delta\phi(\mathbf{r},t) = -4\pi\left(\rho(\mathbf{r},t) + \rho_{\mathrm{jm}}(\mathbf{r})\right), \qquad (13)$$

$$\rho(\mathbf{r},t) = -\sum_{n\mathbf{k}} f_{n\mathbf{k}}|\,u_{n\mathbf{k}}(\mathbf{r},t)|^2, \qquad (14)$$

where $f_{n\mathbf{k}}$ denotes the occupation number. For the exchange-correlation potential V_{XC}, we assume an adiabatic local density approximation[4]. The microscopic electric current density $\mathbf{j}(\mathbf{r},t)$ is given by

$$\mathbf{j}(\mathbf{r},t) = -\mathrm{Re}\left[\sum_{n\mathbf{k}} u_{n\mathbf{k}}^{*}(\mathbf{r},t)\left(-i\nabla + \mathbf{k} + \frac{1}{c}\mathbf{A}(t)\right)u_{n\mathbf{k}}(\mathbf{r},t)\right]. \tag{15}$$

Numerical calculations were carried out using SALMON, an open-source code (https://salmon-tddft.jp/) developed in our group[5]. In SALMON, the TDKS equation is solved in real time, using three-dimensional Cartesian grids to express orbitals and potentials. In our calculation, grid spacings Δx, Δy, Δz, and Δt, are set to 1 Å and 2.5×10^{-3} fs, respectively. The number of k points is chosen from 2×2 to 6×6. The convergence with respect to these parameters was carefully examined. The occupation number is determined by the Fermi-Dirac distribution at a temperature of 300 K.

3. Perturbation theory for the nonlinear optical responses of a SiO_2 thin film

To compare the third-order nonlinearity of the studied metasurface with that of SiO_2 thin films, which are conventionally used in all-optical switches, we estimate the nonlinearity of the latter material using the perturbation theory. We consider a uniform thin film on the *xy* plane located at $0 \le z \le a$ subjected to an incident planar electric field E^{i} whose polarization direction is set parallel to the *x* axis. We assume that a is much smaller than the wavelength of the incident pulse. From here on, only the *x* component of each quantity is considered. We approximate the macroscopic electric current density J flowing the film as

$$J(z,t) = \frac{\theta(z)\theta(a-z)}{a}\tilde{J}(t) = \theta(z)\theta(a-z)\frac{dP(t)}{dt}, \tag{16}$$

where $\tilde{J}(t)$ and $P(t)$ are the 2D electric current density and the macroscopic polarization. In the 2D course graining approximation[1], this current density appears in the Maxwell's equation

(4). We assume the following simplified nonlinear relation between the polarization and the transmitted electric field E^t,

$$P(t) = \chi_1 E^t(t) + \chi_3 \left(E^t(t)\right)^3. \tag{17}$$

By using Eqs. (4), (16) and (17), we obtain

$$E^t(t) = E^i(t) - \frac{2\pi a}{c}\left[\chi_1 \frac{dE^t(t)}{dt} + 3\chi_3 \left(E^t(t)\right)^2 \frac{dE^t(t)}{dt}\right]. \tag{18}$$

We treat χ_3 as the small quantity. Expressing $E^t(t) \approx E_0^t(t) + E_1^t(t)$. We have the zero-th and the first order equations as follows,

$$E_0^t(t) = E^i(t) - \frac{2\pi a \chi_1}{c} \frac{dE_0^t(t)}{dt}, \tag{19}$$

$$E_1^t(t) = -\frac{2\pi a \chi_1}{c} \frac{dE_1^t(t)}{dt} - \frac{6\pi a \chi_3}{c} \left(E_0^t(t)\right)^2 \frac{dE_0^t(t)}{dt}. \tag{20}$$

Now we assume CW waves for $E^i(t)$ and $E_0(t)$ as

$$E^i(t) = F\cos(\omega_i t), \tag{21}$$

$$E_0^t(t) = E_c \cos(\omega_i t) + E_s \sin(\omega_i t), \tag{22}$$

Substituting these assumptions into Eq. (19) leads the following solutions.

$$E_c = \frac{F}{1+\alpha^2}, \tag{23}$$

$$E_s = \frac{\alpha F}{1+\alpha^2}, \tag{24}$$

$$\alpha = \frac{2\pi a \chi_1 \omega_i}{c}. \tag{25}$$

In the same way, we express $E_1^t(t)$ as

$$E_1^t(t) = E_{1c}\cos(\omega_i t) + E_{1s}\sin(\omega_i t) + E_{3c}\cos(3\omega_i t) + E_{3s}\sin(3\omega_i t), \tag{26}$$

and obtain the solutions:

$$E_{1c} = -2\alpha \frac{\alpha\beta F^3}{(1+\alpha^2)^3}, \tag{27}$$

$$E_{1s} = (1-\alpha^2)\frac{\alpha\beta F^3}{(1+\alpha^2)^3}, \tag{28}$$

$$E_{3c} = \frac{\alpha(10\alpha^2-6)}{1+9\alpha^2}\frac{\alpha\beta F^3}{(1+\alpha^2)^3}, \tag{29}$$

$$E_{3s} = \frac{3\alpha^4-12\alpha^2+1}{1+9\alpha^2}\frac{\alpha\beta F^3}{(1+\alpha^2)^3}, \tag{30}$$

$$\beta = \frac{3\chi_3}{4\chi_1}. \tag{31}$$

From Eq. (4), the transmitted and reflected electric fields, E^t and E^r, include the same third-order nonlinear signals. Therefore, we estimated the $R_{NL}^{(3)}$ of the SiO_2 thin film with the thickness $a = 3.1$ nm by:

$$R_{NL}^{(3)} = \frac{E_{3c}^2 + E_{3s}^2}{F^2}. \tag{32}$$

We set the physical parameters as follows[6]: χ_1 and χ_3 are set from the zero-th and second refractive indices $n_0 = 1.44$ and $n_2 = 3.2 \times 10^{-16}$ cm^2/W. F is set to 5.34×10^{-4} a.u. which corresponds to $I = 10^{10}$ W/cm^2. The fundamental frequency ω_i is chosen as 0.82 eV corresponding to the wavelength 1500 nm that is used in the communication S band relying on all-optical switches. Using these parameters, we obtain $R_{NL}^{(3)} = 1.43 \times 10^{-13}$.

References

(1) Yamada, S., Noda, M., Nobusada, K. & Yabana, K. Time-dependent density functional theory for interaction of ultrashort light pulse with thin materials. *Phys. Rev. B* **98**, 245147 (2018).

(2) Ekardt, W. Dynamical Polarizability of Small Metal Particles: Self-Consistent Spherical Jellium Background Model. *Phys. Rev. Lett.* **52**, 1925–1928 (1984).

(3) Brack, M. The physics of simple metal clusters: self-consistent jellium model and semiclassical approaches. *Rev. Mod. Phys.* **65**, 677–732 (1993).

(4) Perdew, J. P. & Zunger, A. Self-interaction correction to density-functional approximations for many-electron systems. *Phys. Rev. B* **23**, 5048–5079 (1981).

(5) Noda, M. *et al.* SALMON: Scalable Ab-initio Light–Matter simulator for Optics and Nanoscience. *Comput. Phys. Commun.* **235**, 356–365 (2019).

(6) Boyd, R. W. *Nonlinear Optics 3rd Edition* (Academic Press, 2008).
